# Circulatory bone morphogenetic protein (BMP) 8B is a non-invasive predictive biomarker for the diagnosis of non-alcoholic steatohepatitis (NASH)

**DOI:** 10.1371/journal.pone.0295839

**Published:** 2023-12-21

**Authors:** Nadella Mounika, Angeera Yadav, Parul Kamboj, Sanjay K. Banerjee, Utpal Jyoti Deka, Savneet Kaur, Ramu Adela

**Affiliations:** 1 Department of Pharmacy Practice, NIPER-Guwahati, Sila Katamur, Halugurisuk, Changsari, Dist.Kamrup, Guwahati, Assam, India; 2 Senior Researcher, Translational Health Science and Technology Institute (THSTI), Faridabad, India; 3 Department of Biotechnology, NIPER-Guwahati, Sila Katamur, Halugurisuk, Changsari, Dist.Kamrup, Guwahati, Assam, India; 4 Department of Gastroenterology, Downtown Hospital, GS Rd, Bormotoria, Guwahati, Assam, India; 5 Department of Molecular and Cellular Medicine, Institute of Liver & Biliary Science (ILBS), Vasant Kunj, New Delhi, India; Kaohsiung Medical University Hospital, TAIWAN

## Abstract

**Background:**

Nonalcoholic fatty liver disease (NAFLD) is a complex disease which is characterized by the deposition of fats in the hepatocytes. Further, it progresses to nonalcoholic steatohepatitis (NASH), fibrosis, and hepatocellular carcinoma. The increasing prevalence of NAFLD urges to find the non-invasive predictive biomarkers. In this study, we sought to determine increased BMP8B levels as predictors for the progression of NAFLD.

**Methods:**

In the present cross-sectional study, circulatory BMP8B levels were measured in healthy controls (n = 56), NAFL patients (n = 72) and NASH patients (n = 77) by using an ELISA kit. Human hepatic BMP8B mRNA expression was measured in the liver tissue of control and NASH patients. In addition, BMP8B expression was confirmed by immunohistochemistry analysis. Furthermore, hepatic BMP8B mRNA expression was measured in wild type (WT) mice, WT mice fed with choline deficient high fat diet (WT+CDHF), iNOS (inducible nitric oxide synthase) knockout (iNOS-/-) mice, iNOS-/- fed with CDHF diet (iNOS-/-+CDHF).

**Results:**

Increased circulatory BMP8B levels and BMP8B mRNA expression in hepatic tissue were significantly higher in NASH patients as compared with the control subjects. BMP8B expression was increased parallel to the fibrosis score in the hepatic tissues of NASH patients. It was observed that increased BMP8B levels have shown a significant positive correlation between aspartate aminotransferase (r = 0.31, p = 0.005), alanine aminotransferase (r = 0.23, p = 0.045), APRI (r = 0.30, p = 0.009), and Fib-4 score (r = 0.25, p = 0.036) in NASH patients. BMP8B has maintained a significant association with NASH and shown high sensitivity (92.91%) and specificity (92.73%) in NASH patients. Furthermore, increased BMP8B mRNA expression levels were observed in iNOS-/-+CDHF mice.

**Conclusion:**

Our study findings confirmed that BMP8B increases with the severity of the disease and BMP8B shows potential as a non-invasive predictive biomarker to identify NAFLD progression. However, future studies should investigate circulatory BMP8B levels in a large number of patients and also its impact on liver during NAFLD progression.

## Introduction

Non-alcoholic fatty liver disease (NAFLD) is a prominent liver disease characterized by lipid accumulation in the hepatocytes. It is now becoming an emerging global public health issue and is considered as a widespread chronic liver disease associated with metabolic diseases [1–3]. NAFLD is a spectrum of liver diseases with pathological stages varying from simple hepatic steatosis (lipids accumulation) to fibrosis, through non-alcoholic steatohepatitis (NASH) (fatty liver and inflammation), and eventually leads to cirrhosis and hepatocellular carcinoma [4,5]. Early diagnosis of NAFLD is essential for effective treatment planning to halt its progression [6]. Till now, liver biopsy is the only diagnostic tool considered as the “gold standard” method to confirm or rule out NAFLD/NASH which is an invasive procedure [7–9]. To overcome this, many non-invasive biomarkers have been developed in the recent years [10]. There is a need for non-invasive testing that can address diagnostic and risk stratification challenges in NAFLD development and progression [11]. Therefore, biomarkers that can be used non-invasively for diagnosing and detecting NASH are urgently needed.

The liver closely communicates with adipose tissue [12] and many studies have identified adipokines as key determinants in NAFLD pathogenesis. However, their role as predictors of the disease remains unexplored. One such secreted adipokine which is induced by obesity and a high-fat diet and has been recently identified to induce hepatic steatosis and inflammation in the *in-vitro* and *in-vivo* steatotic models of NAFLD is BMP8B [13]. BMP8B is a bone morphogenetic protein (BMPs), a member of the transforming growth factor β (TGF-β) family [14]. BMP signaling is involved in multiple cellular processes and physiological functions. It is necessary for the early developmental processes of cell growth, apoptosis, and differentiation [15–18]. Recent findings affirmed that the liver is the main target for BMPs and its main function is to maintain iron homeostasis in liver physiology [19]. BMPs form a ligand with TGF-β and exert their specific effects mainly on inflammation and fibrosis [13].

BMP8B expression has been found in mature brown adipocytes [20,21] and hypothalamus tissues [22]. Brown adipose tissue (BAT), which is a hub for BMP8B can also generate nitric oxide (NO) through inducible nitric oxide synthase (iNOS). BMP8B has been studied in the context of its expression in *in-vitro* models and *in-vivo* models [13]. A recent study has shown that elevated BMP8B expression was observed in the hepatocytes of mice and humans [23]. However, the use of circulatory BMP8B levels as a non-invasive biomarker in humans has not yet been analyzed. Therefore, in the present study, we assessed the circulatory levels of BMP8B in NAFLD/NASH patients and their association with the disease development and progression.

## Materials and methods

### Clinical study population

A total of 205 subjects, both male and female aged between 20–70 years were included. The present cross-sectional study was initiated in the month of March 2022 with prior approval from the Institutional Ethics Committee of the Downtown hospital, Guwahati, and the Institute of Liver and Biliary Sciences (ILBS), New Delhi. The written informed consent form was obtained from all the subjects before their involvement in the study. The procedures were followed as per the principles mentioned in the Declaration of Helsinki.

This study consisted of 56 healthy control subjects (Group 1), 72 NAFL patients (Group 2), and 77 NASH patients (Group 3). **Group 1** control subjects had no history of NAFLD (confirmed by ultrasound report), hypertension, T2DM, or any other diseases. Liver function tests, blood glucose levels, and lipid profile reports were normal. **Group 2** NAFL patients were diagnosed by right upper quadrant ultrasound imaging technique and **Group 3** NASH patients were diagnosed using liver stiffness measurement (LSM) (cutoff value ≥8 kPa) and controlled attenuation parameter (CAP) (cutoff value ≥238 dB/m) by fibroscan report or liver biopsy procedure to confirm the presence of NASH phenotypic features such as simple steatosis and inflammation with or without fibrosis. Most of the NASH patients (88.31%) were diagnosed by liver biopsy procedure. Additionally, laboratory findings (liver function tests) were also taken into consideration. We excluded patients with viral hepatitis, autoimmune hepatitis, and any other liver diseases; cardiovascular disease; impaired kidney function; malignancies; history of abdominal surgery and patients with alcohol intake exceeding 20 g/day.

### Data and sample collection

The required information like demographic details (age, gender), anthropometric details (height, weight, BMI), and history of smoking and alcohol consumption were collected from all the subjects from March 2022 to January 2023. All the participants information was kept confidential during or after data collection.

Blood samples were collected from the study participants by venipuncture. After 30 minutes of blood collection, centrifugation was done at 1500g for 15 minutes at 4°C. Finally, the serum samples of the included subjects were collected and stored at -80°C until analysis. All the required laboratory biochemical parameters were measured as per the standard procedures by using an automated biochemistry analyzer **[**RX Daytona+, Randox laboratories, UK]. Non-invasive markers were calculated [24] as follows: a) AST/ALT ratio (aspartate aminotransferase/alanine aminotransferase), b) APRI (AST to Platelet Ratio Index) = [(AST/ AST upper limit of the normal range) X 100]/Platelet Count, c) HSI (hepatic steatosis index) = 8 × (ALT/AST ratio) + BMI (+2, if female; +2, if diabetes mellitus), d) FIB-4 (fibrosis-4) score = Age (years)×AST (U/L)/[PLT (109/L)×ALT1/2 (U/L)], e) NFS (NAFLD fibrosis score) = [−1.675 + 0.037 –age (years) + 0.094 –BMI (kg/m2) + 1.13 × IFG/diabetes (yes = 1,no = 0) + 0.99 × AST/ALT ratio– 0.013 × platelet count (×109/l)– 0.66 × albumin (g/dl)].

Liver tissue samples were collected from NASH patients who underwent liver biopsy (n = 5) and control subjects who were healthy liver donors (n = 4).

### Serum BMP8B measurement

The quantitative sandwich enzyme immunoassay technique was used for measuring BMP8B in human serum samples [CUSABIO, Catalog No. CSBEL002746HU], with intra- and inter-assay precision having coefficients of variation <8.0% and <10.0% respectively for BMP8B. The assay was performed as per the manufacturer’s instructions and the standard curve was generated. The optical density for BMP8B was determined by using a SpectraMax® Multi-Mode Microplate Reader [Molecular Devices, California] at 450nm and subtracting the values obtained at 540nm.

### BMP8B mRNA expression

#### a) RNA isolation from human liver tissue

RNA was isolated from human (n = 4 control subjects; n = 5 NASH patients) liver tissue (approx 100mg each), using TRIzol reagent, after washing with phosphate-buffered saline solution. To approximately 75-100mg of liver tissue, 1 mL of TRIzol reagent was added and homogenized with a sterile pellet pestle probe. 150μl of chloroform was added to it, vortexed, allowed to stand at room temperature for 5 minutes, and centrifuged. The upper colorless aqueous layer which contains RNA was transferred to a new clean tube. To this tube, an equal amount of isopropyl alcohol was added and centrifuged. The supernatant was removed completely and the RNA pellet was resuspended by adding 75% molecular-grade ethanol. Finally, centrifugation was done and the supernatant was removed. The RNA pellet was completely air-dried and then dissolved in 100μL of nuclease-free water.

#### b) Quantification of mRNA and cDNA synthesis

The integrity of RNA was visually observed by focusing on 18S and 28S ribosomal bands on 1% agarose gel. The purity and concentration of RNA were checked by using a Nanodrop spectrophotometer [Epoch™ Multi-Volume Spectrophotometer System, Thermo Scientific, USA]. Next, 1μg of total RNA was used for cDNA synthesis and this process was done by following the protocol of the Revert Aid First Strand cDNA synthesis kit [Thermo Scientific™]. After cDNA synthesis, a real-time polymerase chain reaction (RT-PCR) [QuantStudio™ 5 Real-Time PCR System] was used to check the BMP8B gene expression.

#### c) Primer design and real time-PCR

BMP8B primer design was done by Primer 3 software. cDNA was analyzed using Absolute SYBR™ Green PCR Master Mix (Takara, Japan). The reaction consisted of cDNA, forward primer, reverse primer, SYBR Green, and nuclease-free H_2_O. The samples were run in duplicate with the following cycling parameters: 1 cycle of 95°C for 10 minutes, 40 cycles of 95°C for 15 seconds, 40 cycles of 58°C for 30 seconds, 40 cycles of 72°C for 30 seconds, 1 cycle of 72°C for 5 minutes concluding with 4°C for 2 minutes. The results were analyzed by matched software. Fold change expression of the gene was determined by normalizing to internal control 18S rRNA expression and presented as the 2^-ΔΔCt formula.

### Immunohistochemistry (IHC) analysis

Liver tissue samples were collected from control subjects (n = 4) and NASH patients with fibrosis scores F0 (n = 4), F2 (n = 4), and F3 (n = 4), followed by tissue fixation, and stained with Hematoxylin and Eosin (H&E). BMP8B immunohistochemistry was performed on tissue sections using primary monoclonal rabbit antibodies (CSB-PA050097) and the assay was performed by following the standard procedure. The staining was observed under a microscope and images were taken at 40X.

### Animal study

#### Animal experimentation

The animal experiment was started only after getting the approval from Institutional Animal Ethical Committee of Translational Health Science and Technology Institute (THSTI), Faridabad. Adult male wild-type (WT) (C57BL/6J), and iNOS knockout (iNOS-/-) mice of age 8–12 weeks and weight 20–25 g were obtained from the animal house of THSTI, Faridabad. Animals were acclimatized to surrounding environmental conditions for one week prior to experimental procedures. They were housed in IVC cages (individually ventilated cages) and maintained at an ambient temperature of 22–25 ± 1°C with a 12:12 hr light/dark cycle.

#### Experimental study diets

Choline deficient High-Fat Diet (CDHF) was used for developing the NAFLD and NASH animal model. CDHF diet was purchased from Research Diets, Inc., New Brunswick, NJ (D05010403), and the Chow diet from Altromin International, Gmb H, Lage/Lippe (1324). The CDHF diet was supplemented with 60% Kcal fat.

#### Study design and sample collection

Animals were randomly assigned into the following four experimental groups: (a) WT mice (n = 5); (b) WT+CDHF (n = 5); (c) iNOS-/- mice (n = 5); (d) iNOS-/- +CDHF (n = 5). Among them, the control group mice (WT and iNOS-/-) were fed with a chow diet and the disease group mice (WT+CDHF and iNOS-/-+CDHF) were fed with CDHF diet for about 10 weeks and provided water *ad libitum* for unless otherwise stated. At the end of the study (10^th^ week), animals were kept on fasting for atleast 6 hours, followed by blood collection and plasma separation for analysis. Subsequently, the animals were sacrificed using CO2; the liver was collected and subjected to gross pathological examination, mechanistic study, and histopathological analyses for individual mice of each group. All the samples were stored at -80°C immediately for further use.

#### Gene expression analysis in mice

Briefly, RNA isolation was carried out from the liver tissue of all groups using Trizol Reagent (T9424, Sigma-Aldrich). Quantitative and qualitative assessment of RNA was done by using a NanoDrop spectrophotometer. cDNA synthesis was carried out via reverse transcription and amplified by real-time PCR. The data were normalized with reference gene 18S ribosomal RNA (18S rRNA) expression. The details of primer sequences are given in S1 Table.

### Statistical analysis

Data are expressed as mean ±SD and median [Inter-quartile range (IQR)] for continuous variables with normal distribution and skewed distribution respectively. Categorical data are presented as numbers. Ordinary one-way ANOVA followed by Tukey’s multiple comparison test was applied for parametric data, whereas, Kruskal-Walli’s test followed by Dunn’s multiple comparison was applied for non-parametric data to check the statistical significance between the groups. In order to find out the correlation between BMP8B and other parameters, Spearman’s correlation analysis was performed. Univariate and multivariate binary logistic regression analysis was also performed to find the relation between BMP8B and covariates. Receiver-operating characteristic (ROC) analysis was used to derive the sensitivity and specificity of BMP8B and other non-invasive markers. The statistical significance was set at p<0.05. All statistical analyses were performed using GraphPad prism 8 software, Inc, La Jolla, CA.

## Results

### Clinical and biochemical characteristics of included subjects

A total of 205 patients from three groups (Control, NAFL, and NASH) were included in this study. Interestingly, age, weight, BMI, all biochemical characteristics (except LDL), and non-invasive markers were found to be statistically significant across the groups. Among study population, male patients were more in number when compared to that of female patients. The detailed information related to socio-demographic characteristics, biochemical characteristics and non-invasive markers was represented in Table 1.

**Table 1:** Clinical and biochemical characteristics of included subjects.

Characteristics	Control (n = 56)	NAFL (n = 72)	NASH (n = 77)	p-value
**Socio-demographic characteristics**				
Age (years)	32.27±9.17	42.18±13.53^a^	52.12±12.60^a,b^	**<0.0001**
Gender (M:F), (n)	33:23	55:17	56:21	**-**
BMI (kg/m^2^)	23.01±3.39	26.37 (23.87–30.30)^a^	28.99±5.70^a^	**<0.0001**
Alcohol consumption (n)				**-**
Former	3	11	7	
Current	1	6	4	
Never	52	55	66	
Smoking (n)				**-**
Former	2	8	7	
Current	4	5	3	
Never	50	59	67	
Diabetes (n)	0	9	20	**-**
Other complications (n)	3	16	8	**-**
**Biochemical characteristics**				
Glucose (mg/dl)	82.60 (74–94.69)	87.52 (78.38–99.96)	107 (89.99–127.8)^**a,b**^	**<0.0001**
AST (U/L)	28.35±10.38	34.41 (28.87–53.32)^**a**^	44.67 (35.00–65.00)^**a,b**^	**<0.0001**
ALT (U/L)	23.96 (15.89–32.93)	35.15 (22.00–60.60)^**a**^	43.00 (31.15–62.50)^**a**^	**<0.0001**
ALP (U/L)	74.68±19.70	91.97 (71.96–122.2)^**a**^	104.5 (88.25–150.4)^**a,b**^	**<0.0001**
Albumin (g/dl)	4.57±0.35	4.56±0.37	3.62±0.76^**a,b**^	**<0.0001**
Total protein (g/dl)	7.78±0.55	7.73±0.54	7.47 (6.90–7.99)^**a,b**^	**0.0008**
Total cholesterol (mg/dl)	158.6±24.99	177.4±35.44^**a**^	184.2±38.22^**a**^	**0.0006**
Triglycerides (mg/dl)	94 (68.86–128.5)	118.2 (85.65–170.6)^**a**^	101.7 (72.68–152.9)	**0.01**
HDL (mg/dl)	40.05 (36–48.10)	41.61±8.66	31.62±10.64^**a,b**^	**<0.0001**
LDL (mg/dl)	117.9±38.55	118.8 (94.68–155.2)	122.9±35.75	0.73
Creatinine (mg/dl)	0.90±0.14	0.98±0.18	0.85 (0.68–1.02)^**b**^	**0.04**
Uric acid (mg/dl)	4.70 (3.83–5.80)	5.96±1.38^**a**^	6.13±1.92^**a**^	**<0.0001**
BUN (mg/dl)	6.61±1.81	7.71 (5.74–9.53)	8.18 (5.50–12.70)^**a**^	**0.002**
Platelets (10^9/L)	182.00±64.05	162.90±63.68	130.6±58.48^**a,b**^	**<0.0001**
**Non-invasive markers**				
APRI	0.37 (0.28–0.52)	0.56 (0.39–0.98)^**a**^	1.02 (0.57–1.93)^**a,b**^	**<0.0001**
AST/ALT	1.08 (0.92–1.53)	0.95 (0.76–1.45)	1.19±0.50	0.34
HSI	31.12±4.17	36.62±6.87^**a**^	37.76±7.60^**a**^	**<0.0001**
FIB-4	0.92 (0.66–1.44)	1.54 (1.13–2.29)^**a**^	3.14 (2.02–6.32)^**a,b**^	**<0.0001**
NFS	-2.55±1.19	-1.44±1.41^**a**^	0.33±1.75^**a,b**^	**<0.0001**
BMP8B	7.34 (0.85–15.12)	31.15 (18.59–68.03)^**a**^	149.6±70.55^**a,b**^	**<0.0001**

Data are presented as mean±SD for parametric and median (25^th^-75^th^ percentile) for non-parametric data. Ordinary one-way ANOVA followed by Tukey’s multiple comparison test was applied for parametric data, whereas, Kruskal-Walli’s test followed by Dunn’s multiple comparison was applied for non-parametric data. p-value <0.05 was considered statistically significant. ^a^compared to control group, ^b^compared to NAFL group. **Abbreviations**: BMI: body mass index; AST: aspartate aminotransferase; ALT: alanine aminotransferase; ALP: alkaline phosphatase; HDL: high density lipoprotein; LDL: low density lipoprotein; BUN: blood urea nitrogen; APRI: AST-to-platelet ratio index; HIS: hepatic steatosis index; FIB-4: fibrosis-4; NFS: NAFLD fibrosis score; BMP8B: Bone morphogenetic protein-8B.

### Measurement of serum BMP8B levels

**Fig 1:**
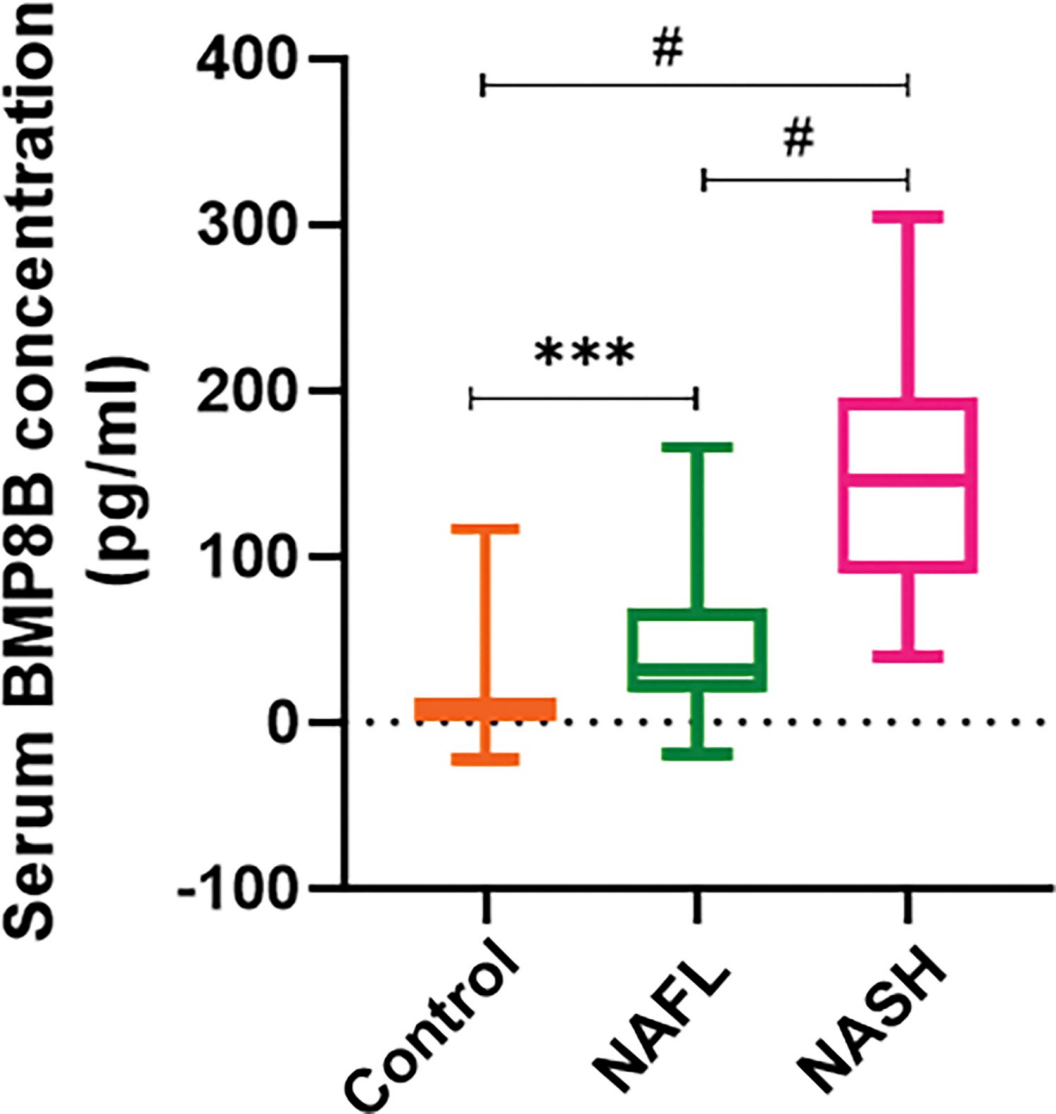
Serum concentration of BMP8B. BMP8B concentration (pg/ml) was measured in serum samples of control (n = 56), NAFL (n = 72), and NASH (n = 77) patients by using an ELISA kit. Kruskal-Wallis One-way ANOVA followed by Dunn’s multiple comparison test was applied to check the statistical significance between the groups. ***p = 0.0001, ^**#**^p < 0.0001. **Abbreviations:** BMP8B: Bone morphogenetic protein-8B; NAFLD: Non-alcoholic fatty liver disease; NASH: Non-alcoholic steatohepatitis; ELISA: enzyme-linked immunosorbent assay.

Serum BMP8B levels were measured in the subjects of control, NAFL, and NASH groups. It was observed that circulatory BMP8B levels were significantly higher in NAFL patients when compared with the control subjects. Correspondingly, circulatory BMP8B levels were found to be significantly higher in NASH patients [mean±SD: 149.6±70.55] than in healthy control individuals [median (25^th^-75^th^ quartile): 7.34 (0.85–15.12)] and NAFL patients [median (25^th^-75^th^ quartile): 31.15 (18.59–68.03)] (Fig 1). Additionally, subgroup analysis was conducted within NAFL group based on the degree of fatty liver (Grade-1 (n = 32), Grade-2 (n = 28), and Grade-3 (n = 12)) and we observed that circulatory BMP8B levels have shown statistically significant differences among the study groups (p = 0.0002). It was also observed that circulatory BMP8B levels are significantly higher in Grade-2 NAFL group (p = 0.03) and Grade-3 NAFL group (p = 0.0002) when compared to the Grade-1 NAFL group (S1 Fig). The correlation was performed and found that a significant positive correlation exists between the degree of fatty liver and BMP8B levels (r = 0.477; p<0.0001) (S1 Fig).

### BMP8B mRNA expression in hepatic tissue

**Fig 2:**
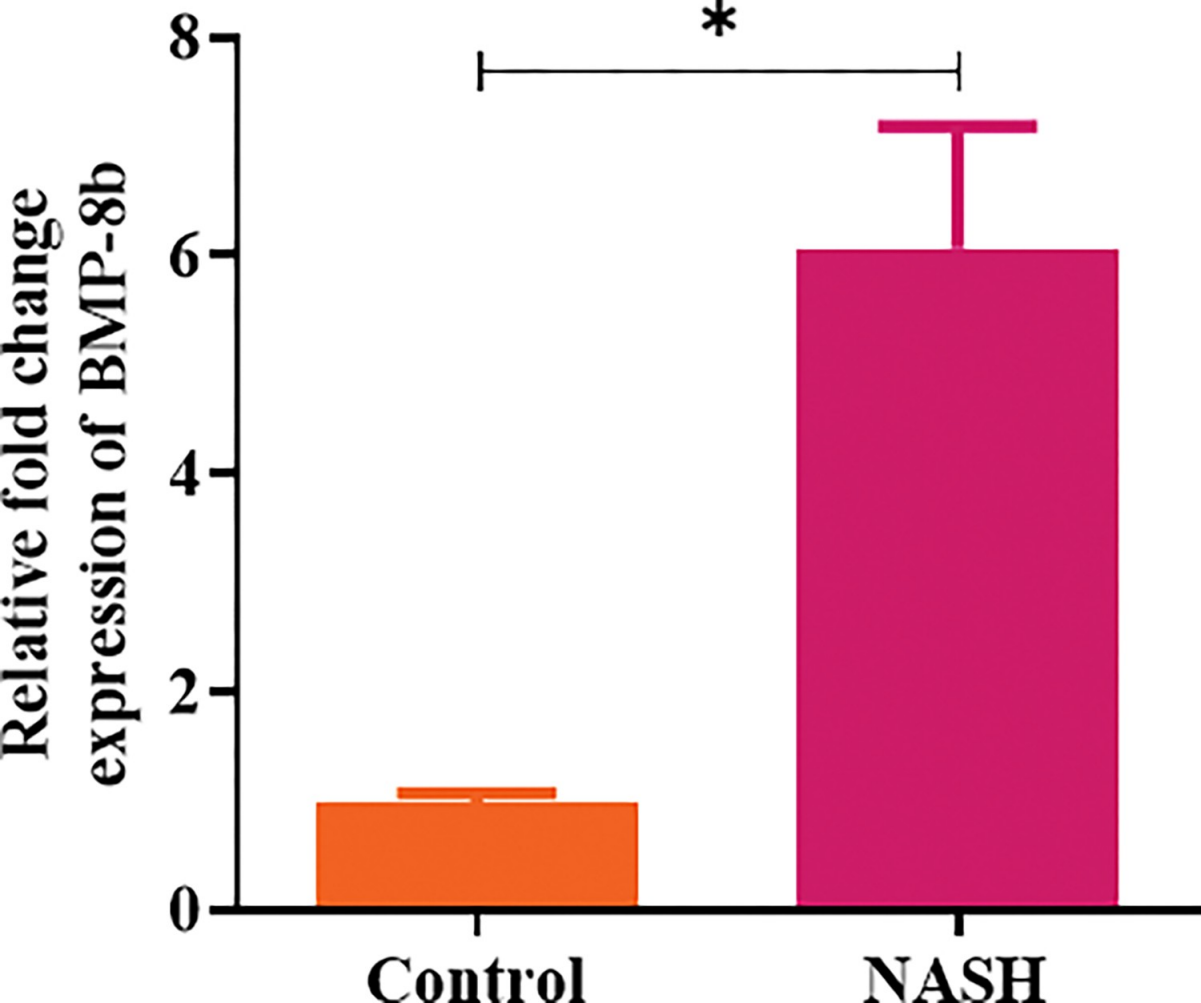
Hepatic BMP8b mRNA expression. RT-PCR was used to measure BMP8b mRNA expression in liver tissues of control subjects (n = 4) and NASH patients (n = 5). Fold change was normalized to 18S rRNA. Data were expressed as mean ± SD. *p < 0.05 was considered statistically significant. **Abbreviations:** BMP8B: Bone morphogenetic protein-8B; NASH: Non-alcoholic steatohepatitis; RT-PCR: Real-time polymerase chain reaction.

**Fig 3:**
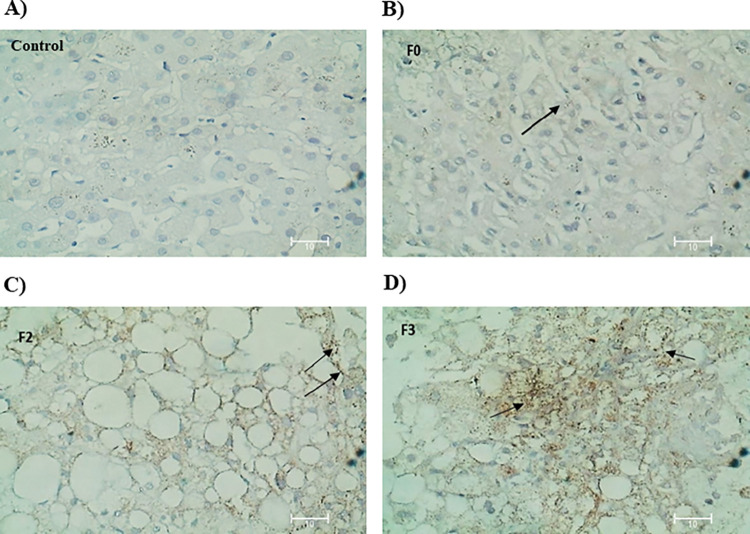
Immunohistochemistry (IHC) analysis of BMP8b expression. IHC analysis was performed in liver tissues of healthy controls and NASH patients. Liver tissues were stained with Hematoxylin and eosin (H&E) staining and then immunostained with BMP8b antibody. A) Control (n = 4), B) F0—No fibrosis (n = 4), C) F2—Moderate fibrosis (n = 4), D) F3—Severe fibrosis (n = 4). The arrows indicate the immune reaction in hepatocytes.

We also analyzed BMP8B mRNA expression in liver biopsies of healthy controls (n = 4) and NASH subjects (n = 5). The results showed a significantly higher mRNA expression of BMP8B in NASH patients than in healthy control individuals (Fig 2). Further, to confirm the expression of BMP8B in the liver tissue of NASH patients based on fibrosis score, IHC analysis was performed. It was observed that the expression of BMP8B was increased in parallel to the fibrosis score. Liver tissue with fibrosis score F0 has shown minimal expression while liver tissue with fibrosis score F3 has shown the highest expression of BMP8B (Fig 3).

### Association of BMP8B with biochemical parameters and non-invasive biomarkers

**Fig 4:**
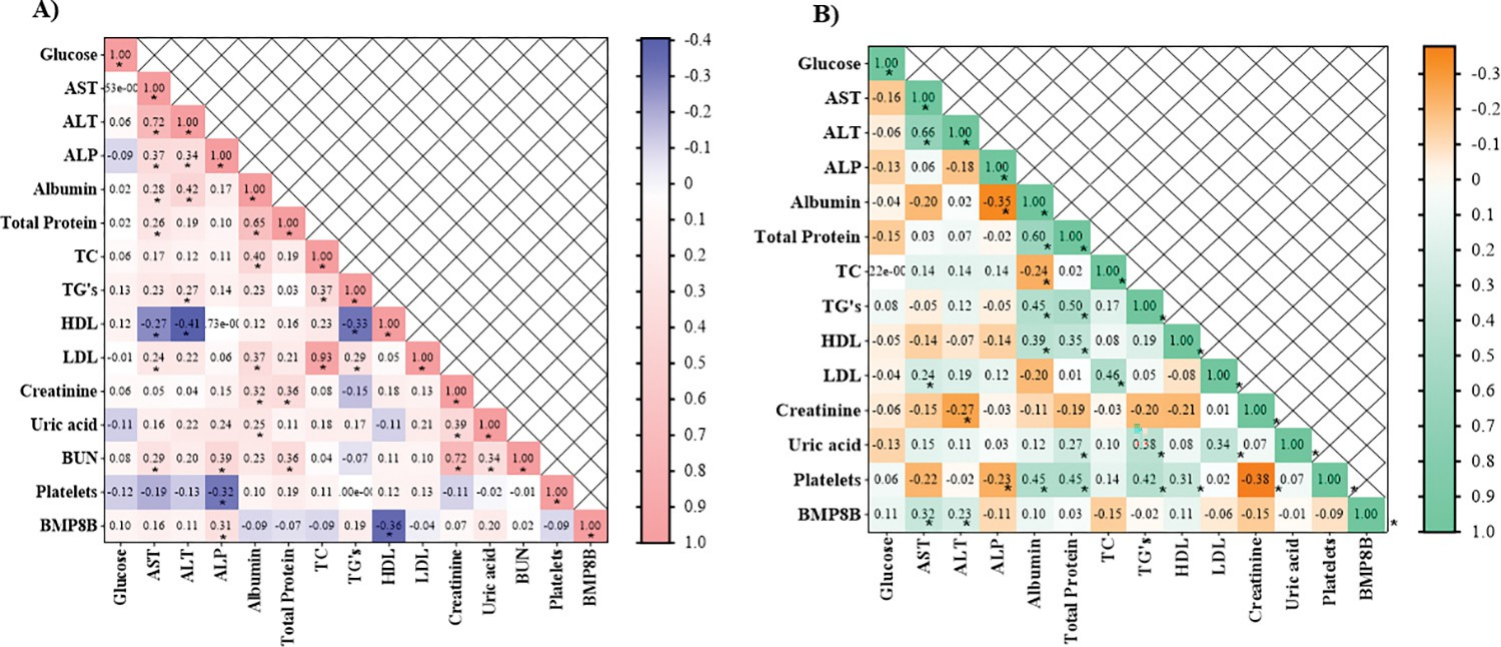
Correlation analysis of BMP8B with biochemical parameters in A) NAFL patients (n = 72), B) NASH patients (n = 77). **Abbreviations:** BMP8B: Bone morphogenetic protein-8B; AST: Aspartate aminotransferase; ALT: Alanine aminotransferase; ALP: Alkaline phosphatase; TC: Total cholesterol; TG: Triglycerides; LDL: low-density lipoprotein; HDL: high density lipoprotein; BUN: Blood urea nitrogen.

We evaluated the correlation of serum BMP8B levels with biochemical characteristics and non-invasive biomarkers of study participants. The results have shown that a significant positive correlation exists between serum BMP8B levels and alkaline phosphatase (ALP) (r = 0.35, p = 0.029), APRI (r = 0.25, p = 0.049), Fib-4 score (r = 0.26, p = 0.037), whereas, a significant negative correlation exists between high-density lipoproteins (HDL) and serum BMP8B levels (r = -0.36, p = 0.009) in NAFL patients. Similarly, a significant positive correlation exists between serum BMP8B levels and aspartate aminotransferase (AST) (r = 0.31, p = 0.005), alanine aminotransferase (ALT) (r = 0.23, p = 0.045), APRI (r = 0.30, p = 0.009), Fib-4 score (r = 0.25, p = 0.036) in NASH patients (Fig 4) (S2 Fig).

### Circulatory BMP8B as an independent predictor

As the circulatory BMP8B levels were significantly higher in NAFLD and NASH patients when compared with the healthy controls, we performed logistic regression analysis separately for the NAFL (Table 2) and NASH (Table 3) groups. Univariate logistic regression analysis revealed that BMP8B along with other significant variables were identified as predictors of NAFL and NASH. In addition, multivariate logistic regression analysis was performed by adjusting some confounding factors that might alter the levels of BMP8B. In model 1 (unadjusted), AST (OR:1.06, 95%CI:1.00–1.11, p = 0.032) and ALP (OR:1.01, 95%CI:1.00–1.03, p = 0.025) were predictors of NAFL, whereas, BMP8B act as an independent predictor of NASH. In model 2, after adjusting for smoking and alcohol history, the results have shown that AST (OR:1.05, 95%CI:1.00–1.11, p = 0.043) and ALP (OR:1.02, 95%CI:1.00–1.04, p = 0.014) have maintained significant association with NAFL, whereas, former alcohol consumers (OR:0.00, 95%CI:0.00–0.77, p = 0.039), current alcohol consumers (OR:0.00, 95%CI:0.00–0.75, p = 0.044), glucose (OR:1.06, 95%CI:1.00–1.12, p = 0.035), AST (OR:1.21, 95%CI:1.02–1.43, p = 0.029), HDL (OR:0.80, 95%CI:0.66–0.97, p = 0.027), and BMP8B (OR:1.04, 95%CI:1.01–1.07, p = 0.001) were predictors of NASH. In model 3, after adjusting for confounding factors such as age, gender, and BMI, the results have shown that BMP8B (OR:1.02, 95%CI:1.00–1.03, p = 0.011) was an independent predictor for NASH but not for NAFL.

**Table 2:** Logistic regression analysis: Control vs NAFL subjects.

Variables	Univariate analysis		Multivariate analysis					
			Model-1		Model-2		Model-3	
	Odds ratio (95% CI)	p-value	Odds ratio (95% CI)	p-value	Odds ratio (95% CI)	p-value	Odds ratio (95% CI)	p-value
Age (yrs) Gender (Male)	1.08 (1.04–1.12) 2.25 (1.05–4.82)	**0.000** **0.036**					1.05 (1.01–1.10) 2.89 (0.89–9.37)	**0.013** 0.076
BMI (kg/m^2^)	1.41 (1.22–1.63)	**0.000**					1.37 (1.14–1.66)	**0.001**
Smoking history (Former)	2.91 (0.58–14.67)	0.194			0.63 (0.08–4.84)	0.659		
Smoking history (Current)	1.04 (0.26–4.08)	0.953			0.17 (0.01–1.91)	0.152		
Alcohol consumption (Former)	3.85 (1.02–14.43)	**0.045**			5.78 (1.18–28.21)	**0.030**		
Alcohol consumption (Current)	5.77 (0.67–49.65)	0.110			3.48 (0.25–48.34)	0.352		
Glucose (mg/dl)	1.01 (0.99–1.02)	0.172						
AST (U/L)	1.08 (1.04–1.12)	**0.000**	1.06 (1.00–1.11)	**0.032**	1.05 (1.00–1.11)	**0.043**	1.05 (0.99–1.11)	0.081
ALT (U/L)	1.03 (1.01–1.06)	**0.001**	1.00 (0.97–1.03)	0.802	1.00 (0.97–1.03)	0.718	1.00 (0.97–1.03)	0.758
ALP (U/L)	1.02 (1.01–1.04)	**0.000**	1.01 (1.00–1.03)	**0.025**	1.02 (1.00–1.04)	**0.014**	1.01 (0.99–1.03)	0.175
Total cholesterol (mg/dl)	1.00 (0.99–1.01)	0.870						
Triglycerides (mg/dl)	1.00 (0.99–1.01)	0.101						
HDL (mg/dl)	0.98 (0.94–1.01)	0.309						
LDL (mg/dl)	1.00 (0.99–1.01)	0.515						
BMP8B (pg/ml)	1.01 (1.00–1.02)	**0.02**	1.00 (0.99–1.02)	0.161	1.00 (0.99–1.02)	0.177	1.00 (0.99–1.01)	0.743

**Model-1:** Unadjusted (considered the variables which are significant in univariate analysis); **Model-2**: Adjusted for alcohol consumption and smoking history; **Model-3:** Adjusted for age, gender, BMI.

**Abbreviations**: BMI: body mass index; AST: aspartate aminotransferase; ALT: alanine aminotransferase; ALP: alkaline phosphatase; HDL: high density lipoprotein; LDL: low density lipoprotein; BMP8B: bone morphogenetic protein-8B.

**Table 3:** Logistic regression analysis: Control vs NASH subjects.

Variables	Univariate analysis		Multivariate analysis					
			Model-1		Model-2		Model-3	
	Odds ratio (95% CI)	p-value	Odds ratio (95% CI)	p-value	Odds ratio (95% CI)	p-value	Odds ratio (95% CI)	p-value
Age (yrs)	1.15 (1.10–1.21)	**0.000**					1.14 (0.95–1.31)	0.281
Gender (Male)	1.85 (0.89–3.86)	0.09					5.66 (0.27–117.08)	0.262
BMI (kg/m^2^)	1.35 (1.20–1.51)	**0.000**					1.62 (0.82–3.20)	0.161
Smoking history (Former)					0.06 (0.00–2.00)	0.120		
Smoking history (Current)					0.44 (0.00–1780.51)	0.849		
Alcohol consumption (Former)					0.00 (0.00–0.77)	**0.039**		
Alcohol consumption (Current)					0.00 (0.00–0.75)	**0.044**		
Glucose (mg/dl)	1.03 (1.01–1.05)	**0.000**	1.01 (0.98–1.03)	0.296	1.06 (1.00–1.12)	**0.035**	1.01 (0.98–1.05)	0.325
AST (U/L)	1.10 (1.05–1.15)	**0.000**	1.09 (0.98–1.20)	0.089	1.21 (1.02–1.43)	**0.029**	1.01 (0.91–1.12)	0.741
ALT (U/L)	1.07 (1.03–1.10)	**0.000**	0.99 (0.94–1.04)	0.750	0.95 (0.87–1.04)	0.304	1.05 (0.96–1.15)	0.253
ALP (U/L)	1.05 (1.03–1.07)	**0.000**	1.03 (0.99–1.06)	0.059	1.02 (0.99–1.06)	0.139	1.03 (0.97–1.08)	0.250
Total cholesterol (mg/dl)	1.00 (0.99–1.01)	0.273						
Triglycerides (mg/dl)	1.00 (0.99–1.00)	0.992						
HDL (mg/dl)	0.89 (0.85–0.93)	**0.000**			0.80 (0.66–0.97)	**0.027**	0.93 (0.80–1.07)	0.356
LDL (mg/dl)	1.00 (0.99–1.01)	0.454						
BMP8B (pg/ml)	1.03 (1.02–1.05)	**0.000**	1.03 (1.01–1.04)	**0.000**	1.04 (1.01–1.07)	**0.001**	1.02 (1.00–1.03)	**0.011**

**Model-1:** Unadjusted (considered the variables which are significant in univariate analysis); **Model-2:** Adjusted for alcohol consumption and smoking history; **Model-3:** Adjusted for age, gender, BMI.

**Abbreviations:** BMI: body mass index; AST: aspartate aminotransferase; ALT: alanine aminotransferase; ALP: alkaline phosphatase; HDL: high density lipoprotein; LDL: low density lipoprotein; BMP8B: bone morphogenetic protein-8B.

### ROC curve analysis of BMP8B

**Fig 5:**
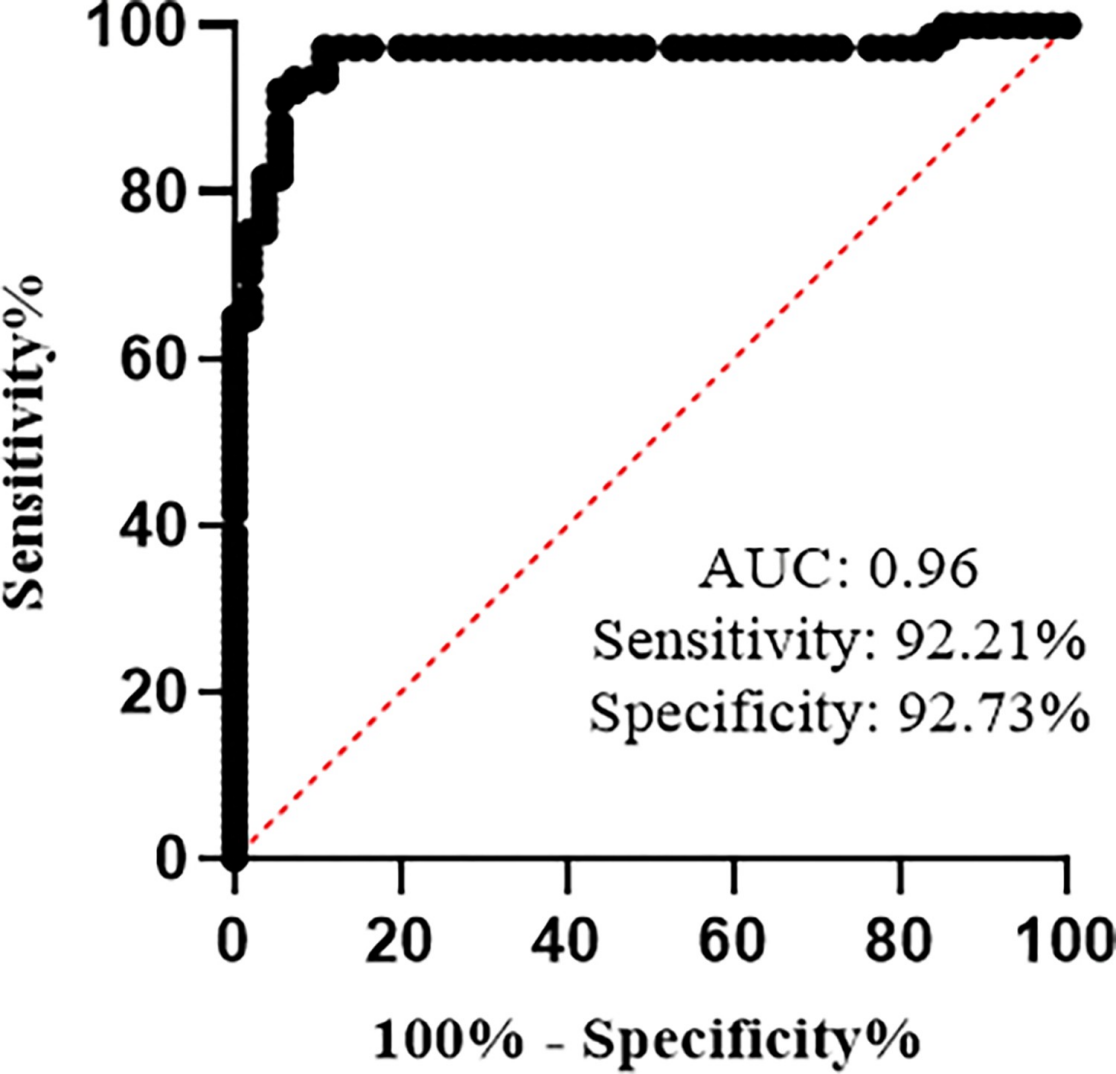
Receiver operator characteristic (ROC) curve analysis of BMP8B. Serum BMP8B concentrations of healthy controls and NASH patients were used for ROC curve analysis. AUC = 0.96; sensitivity = 92.21%; specificity = 92.73%.

After confirming BMP8B as an independent predictor for NASH, we performed ROC curve analysis to discriminate NASH from healthy controls and to determine the diagnostic predictive value of BMP8B for NASH. The area under the ROC curve provided an outstanding result of 0.96 (95% confidence interval: 0.92–0.99; p<0.0001). The cut-off value for BMP8B was found to be >51.59 pg/ml with 92.21% sensitivity (95%CI: 84.02% to 96.38%) and 92.73% specificity (95%CI: 82.74% to 97.14%) respectively (Fig 5). It was found that BMP8B has shown outstanding results when compared with the ROC values of established non-invasive biomarkers (S2 Table).

### BMP8B mRNA expression in liver tissue of mice

**Fig 6:**
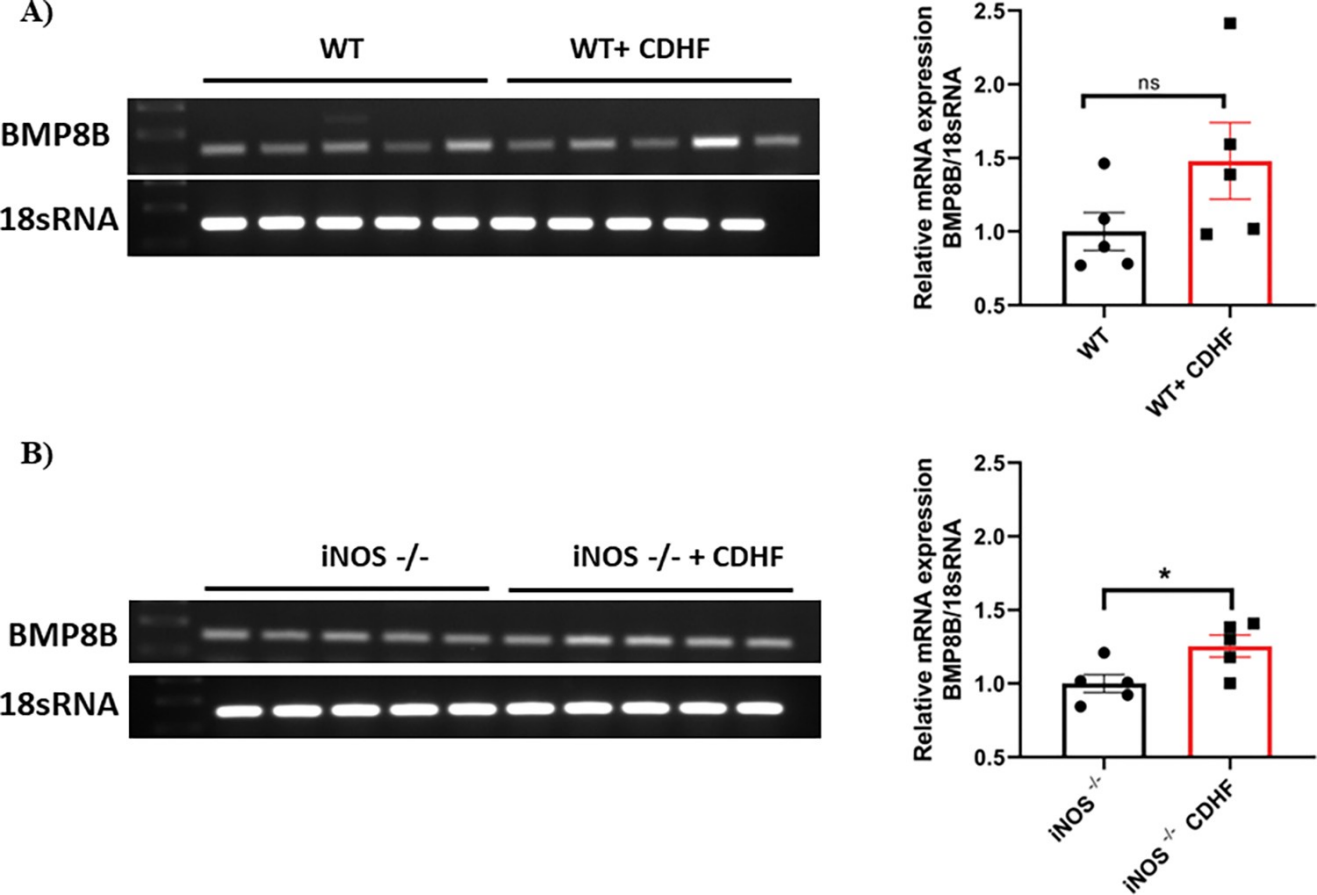
Hepatic BMP8B mRNA expression in mice. Liver tissues were collected from 10 weeks mice fed with chow and CDHF diet. Further, BMP8B mRNA expression was measured by using RT-PCR. Fold change was normalized to 18S rRNA. Data were expressed as mean ± SD. *p<0.05 was considered statistically significant with the corresponding group. A) WT (n = 5) vs WT + CDHF (n = 5). B) iNOS-/- (n = 5) vs iNOS-/- + CDHF (n = 5). **Abbreviations:** BMP8B: Bone morphogenetic protein-8B; CDHF: Choline deficient high fat; WT = wild type; iNOS-/-: inducible nitric oxide synthase knock out.

We explored BMP8B expression in two different mice models of NASH. At first, we have analyzed BMP8B mRNA expression in the liver of WT mice (n = 5) and WT mice fed with choline deficient-high fat (CDHF) diet (WT+CDHF) (n = 5) for 10 weeks and found that BMP8B mRNA expression in WT+CDHF mice was higher but not significant (Fig 6). Secondly, we analyzed BMP8B mRNA expression in the liver of iNOS knock-out (iNOS-/-) mice (n = 5) and iNOS-/- mice fed with choline deficient-high fat (CDHF) diet (iNOS-/-+CDHF) (n = 5) after 10 weeks and found that BMP8B expression in iNOS-/-+CDHF mice was significantly higher (Fig 6).

## Discussion

NAFLD is characterized as the accumulation of fat in the liver and excludes some causes of fat deposits in the liver like significant alcohol consumption, certain medications, etc. [25]. With the increase in the prevalence of NAFLD, there is an increase in the demand for accurate diagnosis and effective therapeutic management of NAFLD/NASH. Furthermore, there is a lack of awareness about the consequences/risk factors of NAFLD development and progression [26] as well as a lack of knowledge on biomarker performance. In order to develop a better therapy, clinical trials should be conducted involving NASH patients [27]. Identification of NASH patients can be done by liver biopsy, which is generally not recommended procedure for recruiting into clinical trials. Therefore, considering all these consequences, there is an urgent need for developing non-invasive biomarkers. In order to develop a precise non-invasive biomarker, research has been emerging in the field of NAFLD. Research studies have demonstrated that the adipokines are involved in NAFLD development and progression by intervening in various activities like steatosis, inflammation, fibrosis, and liver cell death [28,29]. In this study, we opted for BMP8B, an adipokine, and would like to explore the role of BMP8B as a predictive biomarker that might be used to diagnose or detect the progression of NAFLD non-invasively.

Interestingly, this is the first study which reports increased circulatory BMP8B levels in NASH patients when compared with NAFL patients and healthy controls. Among healthy controls and NAFL patients, the circulatory BMP8B levels are more in NAFL patients and found that serum BMP8B levels raised were disease-stage-dependent in humans. Similarly, elevated levels of other BMPs such as BMP4, BMP7, BMP9 were associated with atherosclerosis, hepatic cancer, and metabolic syndrome respectively [30–32]. Furthermore, we checked and observed an upregulated BMP8B expression in the hepatic tissue of NASH patients. Previous studies have reported that the expression of BMPs, especially BMP2 [33], BMP6 [34], and BMP8B [13] was upregulated in the hepatic tissue of NASH patients. Our study findings are consistent with the above study findings and conclude that BMPs might play a major role during the pathophysiology of disease progression. Additionally, immunohistochemistry analysis revealed that expression of BMP8B was increased parallelly to the disease progression. This data suggests that liver tissue is another source of BMP8B during the progressive stages of NAFLD. Usually, BMP8B is not necessary for normal liver function and it is secreted from some other sources. Future studies should investigate on preventing its secretion or activity so that it might be useful to halt NAFLD development and progression. Future studies should provide other mechanisms that may influence BMP8B expression in liver tissue other than steatosis [23]. It was observed that BMP8B acts as a pioneer in NASH progression [13]. As there are a very limited non-invasive biomarkers so far, BMP8B could be one of the biomarkers which shows promising results in discriminating NASH patients from healthy individuals.

Generally, routine blood tests, specifically liver function parameters are used for diagnosis of NAFLD. Elevated levels of liver enzymes are associated with NAFLD pathogenesis. For instance, ALT levels are associated with NAFLD progression [35], AST levels are associated with NASH diagnosis [36], whereas, elevated ALP levels [37] and reduction of HDL levels are associated with NAFLD development [38]. The variations in the levels of liver enzymes may depend on the duration of the disease. However, some studies showed that patients with normal transaminase levels had the histological features of disease progression. Hence, we cannot completely rely on liver enzymes for disease diagnosis [39,40]. Moreover, non-invasive tests such as fibrosis-4 (FIB-4) and NAFLD fibrosis score (NFS), and AST-to-platelet ratio index (APRI) are generally recommended as diagnostic markers by EASL‐EASD‐EASO Clinical Practice Guidelines [41]. Among them, FIB-4 and NFS are mostly used in primary healthcare settings to rule out advanced fibrosis. However, these non-invasive markers need to be calculated based on specific parameters such as age, BMI, platelets, AST, ALT, and albumin.

Notably, these markers have not been used routinely in diagnosing the diseases [10]. Our study findings have shown that serum BMP8B levels were positively correlated with ALP, APRI, and FIB-4 in NAFLD patients, AST, ALT, APRI, and FIB-4 in NASH patients, whereas, a negative correlation was observed with HDL in NAFL patients. These results suggest that BMP8B might be used for diagnosis purposes as that of liver function parameters. It was believed that developing non-invasive biomarkers might be useful for risk stratification. One of the interesting findings of the present study is that circulatory BMP8B levels have maintained a significant association with NASH in multivariate logistic regression analysis. Going further, from our available data, the BMP8B cut-off value of >51.59pg/ml was used to discriminate between healthy control subjects and NASH patients. Previous study has shown that FIB‐4, NFS and APRI can be used to detect the progression of the disease, instead of using liver biopsy [42]. Besides, our study findings revealed that BMP8B has high sensitivity and specificity in detecting NASH patients when compared with the other non-invasive biomarkers that are used in clinical settings in regular intervals. Based on these observations, we conclude that BMP8B can be used as a non-invasive predictive biomarker for NASH and recommend conducting more studies on BMP8B around the world to certify BMP8B as a non-invasive predictive biomarker in the field of NAFLD.

Altogether, our results revealed that BMP8B levels increased with the severity of the disease in both circulatory levels (ELISA method) and in biopsy-proven NASH liver tissue samples (IHC analysis). Further, we investigated whether the same findings of BMP8B upregulation was applicable during disease progression in NASH animal models. In the present study, we have chosen two animal models of NASH in which CDHF diet was used to develop the phenotypes of NASH such as hepatic fat accumulation, lobular inflammation, and hepatocyte ballooning [43]. During the development of NASH phenotypes, hepatic stellate cells (HSCs) will get activated which thereby implicates the occurrence of BMP8B [44,45]. Typically, iNOS expression has not been shown in ordinary conditions. Most of the studies have indicated that iNOS (inducible nitric oxide synthase) and NO (nitric oxide) play a crucial role in disease development particularly, which are associated with inflammation like NASH [46]. Although the absence of iNOS is expected to observe less NASH phenotype, the reality is different. A previous study has shown that iNOS**-/-** mice has more tendency to fat accumulation in the liver when compared to wild-type (WT) mice [47]. As expected, our unpublished data showed that iNOS-/- mice showed more NAFLD phenotype i.e., higher fat accumulation and inflammation compared to wild type mice. Therefore, we have checked the BMP8B gene expression in both wild-type and iNOS-/- NAFLD mice models to understand if BMP8B expression depends on NAFLD progression and complexity. In our data, we have observed changes in BMP8B levels in the WT+CDHF diet group compared to the WT group. However, changes were not significant. This might be due to protection from iNOS-derived NO production. As expected, iNOS-/-+CDHF mice model showed severe phenotypic changes along with higher BMP8B levels in the liver when compared to the WT+CDHF mice model. Together, the human and animal data showed that the liver is another source of BMP8B. In addition, BMP8B could induce lipid accumulation, activation of NF-κB, and upregulation of pro-inflammatory gene expression in hepatocytes [23]. Moreover, BMP8B could be a molecular therapeutic target by preventing its induction without altering the beneficial effects caused by some other genes. Future studies are warranted with detailed insight into the molecular mechanisms involved behind the role of elevated BMP8B levels in NASH conditions and its impact on hepatic steatosis and inflammation.

Moreover, the limitations of the current study included relatively a smaller number of participants in the three study groups. We have considered either FibroScan or liver biopsy procedure for NASH diagnosis. Measurement of BMP8B levels and their association with biochemical parameters and non-invasive biomarkers was not performed in all the stages of NAFLD progression. Since this is a single timepoint study, it does not address causality relationship. The obtained results could be well performed in different ethnic backgrounds needs further research. However, future studies focusing on both hypothesis driven approaches and machine-learning approaches should be implemented.

## Conclusion

Our study findings revealed that BMP8B could be used as a potential non-invasive predictive biomarker to identify NAFLD progression. The elevated serum BMP8B levels observed in NAFLD patients were due to an increased expression of BMP8B in the liver. In addition, BMP8B could effectively discriminate NASH patients with a good sensitivity and specificity in our study population when compared with the other non-invasive biomarkers. We believe that further investigations in large cohorts of NAFLD and NASH patients is very important to focus on the role of BMP8B as a predictive non-invasive biomarker.

## Supporting information

S1 Fig Data related to different grades of NAFL group: a) Serum BMP8B levels within different grades of NAFL group. b) Correlation between different grades of NAFL group and BMP8B levels (r = 0.477; p<0.0001).
(DOCX)

S2 Fig Correlation analysis of BMP8B with non-invasive biomarkers in A) NAFLD patients (n = 72), B) NASH patients (n = 77). (DOCX)

S1 Table Details of human and mice BMP8B primer. (DOCX)

S2 Table ROC curve analysis of non-invasive biomarkers in NAFLD and NASH patients. (DOCX)

S1 File S1 Raw image. (TIF)

## References

[1] Younossi Z, Anstee QM, Marietti M, Hardy T, Henry L, Eslam M, George J, Bugianesi E. Global burden of NAFLD and NASH: trends, predictions, risk factors and prevention. Nat Rev Gastroenterol Hepatol. 2018;15(1):11–20. doi: 10.1038/nrgastro.2017.109 28930295

[2] Sanyal AJ; American Gastroenterological Association. AGA technical review on nonalcoholic fatty liver disease. Gastroenterology. 2022;123(5):1705–25. 10.1053/gast.2002.36572 12404245

[3] Anstee QM, Targher G, Day CP. Progression of NAFLD to diabetes mellitus, cardiovascular disease or cirrhosis. Nat Rev Gastroenterol Hepatol. 2013;10(6):330–44. doi: 10.1038/nrgastro.2013.41 23507799

[4] Dyson JK, Anstee QM, McPherson S. Non-alcoholic fatty liver disease: a practical approach to treatment. Frontline Gastroenterol. 2014;5(4):277–286. doi: 10.1136/flgastro-2013-100404 25285192 PMC4173737

[5] Anstee QM, McPherson S, Day CP. How big a problem is non-alcoholic fatty liver disease? BMJ. 2011;343:d3897 doi: 10.1136/bmj.d3897 21768191

[6] Pardhe BD, Shakya S, Bhetwal A, et al. Metabolic syndrome and biochemical changes among non-alcoholic fatty liver disease patients attending a tertiary care hospital of Nepal. BMC Gastroenterol. 2018;18(1):109. doi: 10.1186/s12876-018-0843-6 29980170 PMC6035472

[7] Obika M, Noguchi H. Diagnosis and evaluation of nonalcoholic fatty liver disease. Exp Diabetes Res. 2012;2012:145754. doi: 10.1155/2012/145754 22110476 PMC3205741

[8] Nalbantoglu IL, Brunt EM. Role of liver biopsy in nonalcoholic fatty liver disease. World J Gastroenterol. 2014;20(27):9026–37. doi: 10.3748/wjg.v20.i27.9026 25083076 PMC4112884

[9] Singh SP, Barik RK. NonInvasive Biomarkers in Nonalcoholic Fatty Liver Disease: Are We There Yet? J Clin Exp Hepatol. 2020;10(1):88–98. doi: 10.1016/j.jceh.2019.09.006 32025168 PMC6995889

[10] Castera L, Friedrich-Rust M, Loomba R. Noninvasive assessment of liver disease in patients with nonalcoholic fatty liver disease. Gastroenterology. 2019;156(5):1264–81. doi: 10.1053/j.gastro.2018.12.036 30660725 PMC7505052

[11] Tincopa MA, Loomba R. Non-invasive diagnosis and monitoring of non-alcoholic fatty liver disease and non-alcoholic steatohepatitis. Lancet Gastroenterol Hepatol. 2023;8(7):660–670. doi: 10.1016/S2468-1253(23)00066-3 37060912

[12] Wree A, Kahraman A, Gerken G, Canbay A. Obesity affects the liver—the link between adipocytes and hepatocytes. Digestion. 2011;83(1–2):124–33. doi: 10.1159/000318741 21042023

[13] Vacca M, Leslie J, Virtue S, et al. Bone morphogenetic protein 8B promotes the progression of non-alcoholic steatohepatitis. Nat Metab. 2020;2(6):514–531. doi: 10.1038/s42255-020-0214-9 32694734 PMC7617436

[14] Chen Di, Zhao Ming & Mundy Gregory R. Bone Morphogenetic Proteins. Growth Factors. 2004;22(4):233–41. doi: 10.1080/08977190412331279890 15621726

[15] Hemmati-Brivanlou A., Thomsen G.H. Ventral mesodermal patterning in Xenopus embryos: expression patterns and activities of BMP-2 and BMP-4. Dev Genet. 1995;17(1):78–89. doi: 10.1002/dvg.1020170109 7554498

[16] Zou H., Niswander L. Requirement for BMP signaling in interdigital apoptosis and scale formation. Science. 1996;272(5262):738–41. doi: 10.1126/science.272.5262.738 8614838

[17] Stewart A., Guan H., Yang K. BMP-3 promotes mesenchymal stem cell proliferation through the TGF-beta/activin signaling pathway. J Cell Physiol. 2010;223(3):658–66. doi: 10.1002/jcp.22064 20143330

[18] Kobayashi T, Lyons KM, McMahon AP, Kronenberg HM. BMP signaling stimulates cellular differentiation at multiple steps during cartilage development. Proc Natl Acad Sci U S A. 2005;102(50):18023–7. doi: 10.1073/pnas.0503617102 16322106 PMC1312369

[19] Herrera B, Addante A, Sánchez A. BMP Signalling at the Crossroad of Liver Fibrosis and Regeneration. Int J Mol Sci. 2017;19(1):39. doi: 10.3390/ijms19010039 29295498 PMC5795989

[20] Whittle A.J., Carobbio S., Martins L. et al. Bmp8b increases brown adipose tissue thermogenesis through both central and peripheral actions. Cell. 2012;149(4):871–85. doi: 10.1016/j.cell.2012.02.066 22579288 PMC3383997

[21] Martins L., Seoane-Collazo P., Contreras C. et al. A functional link between ampk and orexin mediates the effect of bmp8b on energy balance. Cell Rep. 2016;16(8):2231–2242. doi: 10.1016/j.celrep.2016.07.045 27524625 PMC4999418

[22] Contreras C., Gonzalez F., Ferno J., Dieguez C., Rahmouni K., Nogueiras R., Lopez M. The brain and brown fat. Ann. Med. 2015;47(2):150–68. doi: 10.3109/07853890.2014.919727 24915455 PMC4438385

[23] Mahli A, Seitz T, Beckröge T, et al. Bone Morphogenetic Protein-8B Expression is Induced in Steatotic Hepatocytes and Promotes Hepatic Steatosis and Inflammation In Vitro. Cells. 2019;8(5):457. doi: 10.3390/cells8050457 31096638 PMC6562647

[24] Kahl S., Straßburger K., Nowotny B., et al. Comparison of liver fat indices for the diagnosis of hepatic steatosis and insulin resistance. PLoS ONE. 2014;9(4). doi: 10.1371/journal.pone.0094059 24732091 PMC3986069

[25] Puri P, Sanyal AJ. Nonalcoholic fatty liver disease: Definitions, risk factors, and workup. Clin Liver Dis. 2012;1(4):99–103. doi: 10.1002/cld.81 31186860 PMC6499283

[26] Lindenmeyer CC, McCullough AJ. The natural history of nonalcoholic fatty liver disease-an evolving view. Clin Liver Dis. 2018;22(1):11–21. doi: 10.1016/j.cld.2017.08.003 29128051 PMC6130315

[27] Vali Yasaman, Lee Jenny, Boursier Jerome, Petta Salvatore, Wonders Kristy, et al. Biomarkers for staging fibrosis and non-alcoholic steatohepatitis in non-alcoholic fatty liver disease (the LITMUS project): a comparative diagnostic accuracy study. Lancet Gastroenterol Hepatol. 2023:S2468-1253(23)00017–1. 10.1016/S2468-1253(23)00017-1 36958367

[28] Tilg H, Hotamisligil G. Nonalcoholic fatty liver disease: cytokine-adipokine interplay and regulation of insulin resistance. Gastroenterology. 2006;131(3):934–45. doi: 10.1053/j.gastro.2006.05.054 16952562

[29] Polyzos SA, Kountouras J, Zavos C. Nonalcoholic fatty liver disease: the pathogenetic roles of insulin resistance and adipocytokines. Curr Mol Med. 2009;9(3):299–314. doi: 10.2174/156652409787847191 19355912

[30] Son JW, Jang EH, Kim MK, Baek KH, Song KH, Yoon KH, et al. Serum BMP-4 levels in relation to arterial stiffness and carotid atherosclerosis in patients with type 2 diabetes. Biomark Med. 2011;5(6):827–35. doi: 10.2217/bmm.11.81 22103619

[31] Xu X, Li X, Yang G, Li L, Hu W, Zhang L, et al. Circulating bone morphogenetic protein-9 in relation to metabolic syndrome and insulin resistance. Sci Rep. 2017;7(1):17529. doi: 10.1038/s41598-017-17807-y 29235531 PMC5727514

[32] Schmierer B, Hill CS. TGFbeta-SMAD signal transduction: molecular specificity and functional flexibility. Nat Rev Mol Cell Biol. 2007;8(12):970–82. doi: 10.1038/nrm2297 18000526

[33] Marañón P., Fernández-García C.E., Isaza S.C. et al. Bone morphogenetic protein 2 is a new molecular target linked to non-alcoholic fatty liver disease with potential value as non-invasive screening tool. Biomark Res. 2022;10(1):35. doi: 10.1186/s40364-022-00383-3 35614516 PMC9131682

[34] Arndt S, Wacker E, Dorn C, Koch A, Saugspier M, Thasler WE, et al. Enhanced expression of BMP6 inhibits hepatic fibrosis in non-alcoholic fatty liver disease. Gut. 2015;64(6):973–81. doi: 10.1136/gutjnl-2014-306968 25011936

[35] Ulasoglu C, Enc FY, Kaya E, Yilmaz Y. Characterization of patients with biopsy-proven non-alcoholic fatty liver disease and normal aminotransferase levels. J Gastrointestin Liver Dis. 2019;28(4):427–431. doi: 10.15403/jgld-293 31826068

[36] Hadizadeh F, Faghihimani E, Adibi P. Nonalcoholic fatty liver disease: Diagnostic biomarkers. World J Gastrointest Pathophysiol. 2017;8(2):11–26. doi: 10.4291/wjgp.v8.i2.11 28573064 PMC5437499

[37] Zhou YJ, Zou H, Zheng JN, Zou TT, Vitale A, Miele L, Van Poucke S, Liu WY, Shen S, Zhang DC, Shi KQ, Zheng MH. Serum alkaline phosphatase, a risk factor for non-alcoholic fatty liver, but only for women in their 30s and 40s: evidence from a large cohort study. Expert Rev Gastroenterol Hepatol. 2017;11(3):269–276. doi: 10.1080/17474124.2017.1283984 28095261

[38] Mansour-Ghanaei R, Mansour-Ghanaei F, Naghipour M, Joukar F. Biochemical markers and lipid profile in nonalcoholic fatty liver disease patients in the PERSIAN Guilan cohort study (PGCS), Iran. J Family Med Prim Care. 2019;8(3):923–928. doi: 10.4103/jfmpc.jfmpc_243_18 31041226 PMC6482810

[39] Barusrux S, Nanok C, Puthisawas W, et al. Viral hepatitis B, C infection and genotype distribution among cholangiocarcinoma patients in northeast Thailand. Asian Pac J Cancer Prev. 2012;13(Suppl):83–7. 23480769

[40] Lee CH, Hsieh SY, Chang CJ, et al. Comparison of clinical characteristics of combined hepatocellular-cholangiocarcinoma and other primary liver cancers. J Gastroenterol Hepatol. 2013;28:122–7. doi: 10.1111/j.1440-1746.2012.07289.x 23034166

[41] European Association for the Study of the L, European Association for the Study of D, European Association for the Study of O. EASL‐EASD‐EASO Clinical Practice Guidelines for the management of non‐alcoholic fatty liver disease. J Hepatol. 2016;64(6):1388‐1402. doi: 10.1016/j.jhep.2015.11.004 27062661

[42] Lee J, Vali Y, Boursier J, Spijker R, Anstee QM, Bossuyt PM, Zafarmand MH. Prognostic accuracy of FIB-4, NAFLD fibrosis score and APRI for NAFLD-related events: A systematic review. Liver Int. 2021;41(2):261–270. doi: 10.1111/liv.14669 32946642 PMC7898346

[43] Lau J.K.; Zhang X.; Yu J. Animal models of non-alcoholic fatty liver disease: Current perspectives and recent advances. J Pathol. 2017;241(1):36–44. doi: 10.1002/path.4829 27757953 PMC5215469

[44] Hardy T., Anstee Q. M. & Day C. P. Nonalcoholic fatty liver disease: new treatments. Curr. Opin. Gastroenterol. 2015;31(3):175–83. doi: 10.1097/MOG.0000000000000175 25774446 PMC4482455

[45] Brunt E. M. et al. Nonalcoholic fatty liver disease. Nat Rev Dis Primers. 2015;1:15080. doi: 10.1038/nrdp.2015.80 27188459

[46] Wei CL, Hon WM, Lee KH, Khoo HE. Temporal expression of hepatic inducible nitric oxide synthase in liver cirrhosis. World J Gastroenterol. 2005;11(3):362–7. doi: 10.3748/wjg.v11.i3.362 15637745 PMC4205338

[47] Kanuri BN, Kanshana JS, Rebello SC, Pathak P, Gupta AP, Gayen JR, Jagavelu K, Dikshit M. Altered glucose and lipid homeostasis in liver and adipose tissue pre-dispose inducible NOS knockout mice to insulin resistance. Sci Rep. 2017;7:41009. doi: 10.1038/srep41009 28106120 PMC5247703

