# Supplementary material for: Circulatory bone morphogenetic protein (BMP) 8B is a non-invasive predictive biomarker for the diagnosis of non-alcoholic steatohepatitis (NASH)
Source: PLoS One. 2023 Dec 21;18(12):e0295839. doi: 10.1371/journal.pone.0295839 (PMC10734958; doi:10.1371/journal.pone.0295839)
Supplement: S1 Fig — Data related to different grades of NAFL group: a) Serum BMP8B levels within different grades of NAFL group. b) Correlation between different grades of NAFL group and BMP8B levels (r = 0.477; p<0.0001). (DOCX) [file pone.0295839.s001.docx]

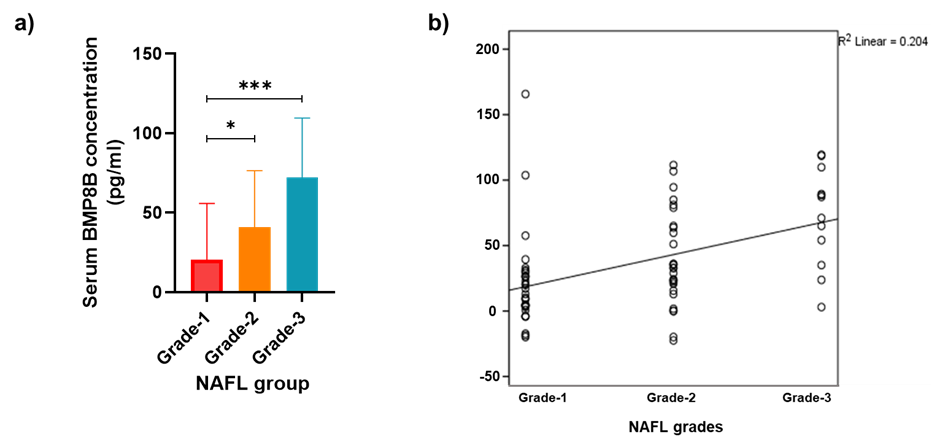


**S1 Fig:** **Data related to different grades of NAFL group:** a) Serum BMP8B levels within different grades of NAFL group. **b)** Correlation between different grades of NAFL group and BMP8B levels (r=0.477; p<0.0001).
