# Supplementary material for: Circulatory bone morphogenetic protein (BMP) 8B is a non-invasive predictive biomarker for the diagnosis of non-alcoholic steatohepatitis (NASH)
Source: PLoS One. 2023 Dec 21;18(12):e0295839. doi: 10.1371/journal.pone.0295839 (PMC10734958; doi:10.1371/journal.pone.0295839)
Supplement: S2 Fig — (DOCX) [file pone.0295839.s002.docx]

**A)**

**B)**

**S2 Fig:** Correlation analysis of BMP8B with non-invasive biomarkers in A) NAFLD patients (n=72), B) NASH patients (n=77).
