# Supplementary material for: Circulatory bone morphogenetic protein (BMP) 8B is a non-invasive predictive biomarker for the diagnosis of non-alcoholic steatohepatitis (NASH)
Source: PLoS One. 2023 Dec 21;18(12):e0295839. doi: 10.1371/journal.pone.0295839 (PMC10734958; doi:10.1371/journal.pone.0295839)
Supplement: S1 Table — (DOCX) [file pone.0295839.s003.docx]

**S1 Table:** **Details of human and mice BMP8B primer**

| **Gene** | **Forward primer** | **Reverse primer** |
| --- | --- | --- |
| **BMP8B (human)** | AGGTGGCTTCCTTATCTGCG | ATGTGCCAACTCTGCTTCGT |
| **BMP8B (mice)** | CTATGCAGGCCCTGGTACAT | AGGCCTGGACTACCATGTTG |
| **18S rRNA** | GCAATTATTCCCCATGAACG | GGCCTCACTAAACCATCCAA |
