# Supplementary material for: Circulatory bone morphogenetic protein (BMP) 8B is a non-invasive predictive biomarker for the diagnosis of non-alcoholic steatohepatitis (NASH)
Source: PLoS One. 2023 Dec 21;18(12):e0295839. doi: 10.1371/journal.pone.0295839 (PMC10734958; doi:10.1371/journal.pone.0295839)
Supplement: S2 Table — (DOCX) [file pone.0295839.s004.docx]

**S2 Table:** **ROC curve analysis of non-invasive biomarkers in NAFLD and NASH patients**

| **S.No.** | **Non-invasive markers** | **AUC value** | **95% CI** | **Sensitivity** | **Specificity** | **p-value** |
| --- | --- | --- | --- | --- | --- | --- |
| **Control vs NAFLD** | | | | | | |
| 1. | APRI | 0.71 | 0.62-0.80 | 56.94 | 75.00 | **<0.0001** |
| 2. | AST/ALT | 0.56 | 0.47-0.66 | 45.07 | 75.00 | 0.17 |
| 3. | HSI | 0.76 | 0.68-0.84 | 61.97 | 76.79 | **<0.0001** |
| 4. | FIB-4 | 0.72 | 0.64-0.81 | 70.42 | 71.43 | **<0.0001** |
| 5. | NFS | 0.72 | 0.63-0.80 | 66.20 | 69.64 | **<0.0001** |
| **Control vs NASH** | | | | | | |
| 6. | APRI | 0.84 | 0.77-0.90 | 79.22 | 75 | **<0.0001** |
| 7. | AST/ALT | 0.50 | 0.40-0.60 | 52.63 | 57.14 | 0.97 |
| 8. | HSI | 0.78 | 0.71-0.86 | 68.42 | 76.79 | **<0.0001** |
| 9. | FIB-4 | 0.87 | 0.81-0.93 | 82.89 | 75.00 | **<0.0001** |
| 10. | NFS | 0.90 | 0.85-0.95 | 82.89 | 82.14 | **<0.0001** |

Bold indicates p-value was statistically significant.
