# Supplementary figures and images for: Circulatory bone morphogenetic protein (BMP) 8B is a non-invasive predictive biomarker for the diagnosis of non-alcoholic steatohepatitis (NASH)
Source: PLoS One. 2023 Dec 21;18(12):e0295839. doi: 10.1371/journal.pone.0295839 (PMC10734958; doi:10.1371/journal.pone.0295839)

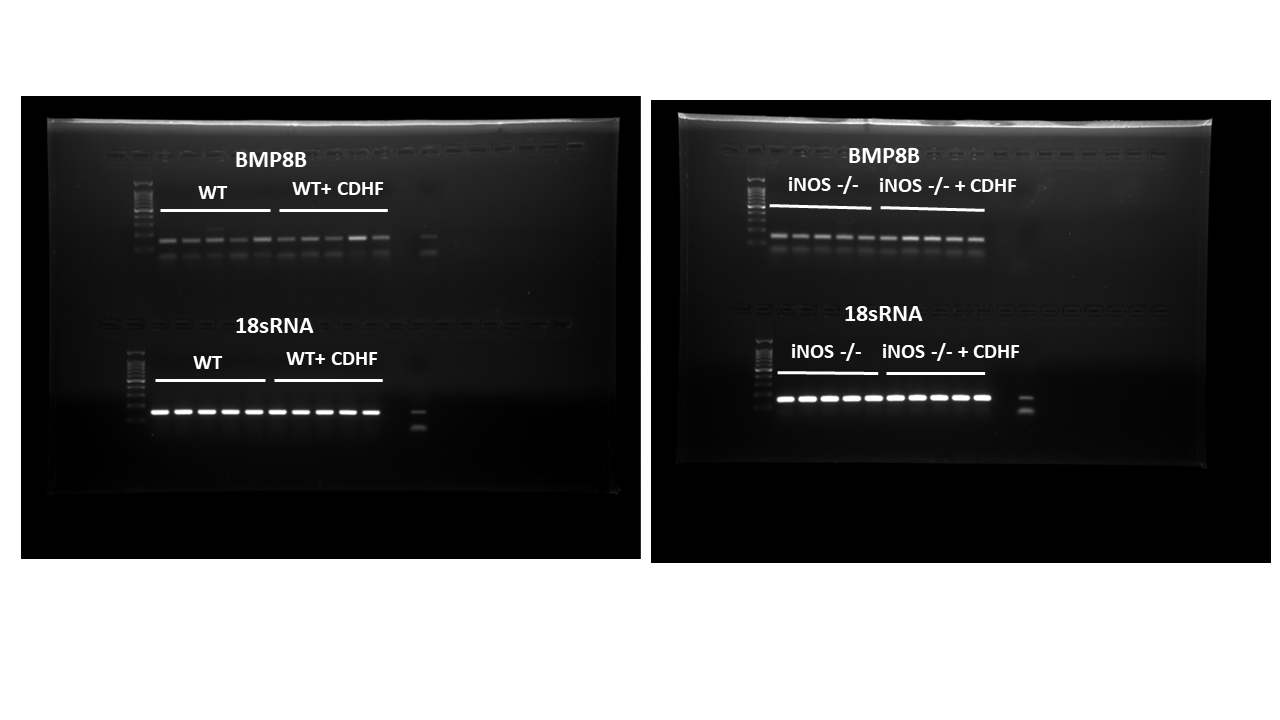

Supplement: S1 File — (TIF) [file pone.0295839.s005.tif]
